# RT-Transformer: retention time prediction for metabolite annotation to assist in metabolite identification

**DOI:** 10.1093/bioinformatics/btae084

**Published:** 2024-02-24

**Authors:** Jun Xue, Bingyi Wang, Hongchao Ji, WeiHua Li

**Affiliations:** School of Information Science and Engineering, Yunnan University, Kunming, Yunnan 650500, China; Shenzhen Branch, Guangdong Laboratory for Lingnan Modern Agriculture, Genome Analysis Laboratory of the Ministry of Agriculture and Rural Affairs, Agricultural Genomics Institute at Shenzhen, Chinese Academy of Agricultural Sciences, Shenzhen, Guangdong 518120, China; Yunnan Police College, Kunming, Yunnan 650223, China; Key Laboratory of Smart Drugs Control (Yunnan Police College), Ministry of Education, Kunming, Yunnan 650223, China; Shenzhen Branch, Guangdong Laboratory for Lingnan Modern Agriculture, Genome Analysis Laboratory of the Ministry of Agriculture and Rural Affairs, Agricultural Genomics Institute at Shenzhen, Chinese Academy of Agricultural Sciences, Shenzhen, Guangdong 518120, China; School of Information Science and Engineering, Yunnan University, Kunming, Yunnan 650500, China

## Abstract

**Motivation:**

Liquid chromatography retention times prediction can assist in metabolite identification, which is a critical task and challenge in nontargeted metabolomics. However, different chromatographic conditions may result in different retention times for the same metabolite. Current retention time prediction methods lack sufficient scalability to transfer from one specific chromatographic method to another.

**Results:**

Therefore, we present RT-Transformer, a novel deep neural network model coupled with graph attention network and 1D-Transformer, which can predict retention times under any chromatographic methods. First, we obtain a pre-trained model by training RT-Transformer on the large small molecule retention time dataset containing 80 038 molecules, and then transfer the resulting model to different chromatographic methods based on transfer learning. When tested on the small molecule retention time dataset, as other authors did, the average absolute error reached 27.30 after removing not retained molecules. Still, it reached 33.41 when no samples were removed. The pre-trained RT-Transformer was further transferred to 5 datasets corresponding to different chromatographic conditions and fine-tuned. According to the experimental results, RT-Transformer achieves competitive performance compared to state-of-the-art methods. In addition, RT-Transformer was applied to 41 external molecular retention time datasets. Extensive evaluations indicate that RT-Transformer has excellent scalability in predicting retention times for liquid chromatography and improves the accuracy of metabolite identification.

**Availability and implementation:**

The source code for the model is available at https://github.com/01dadada/RT-Transformer. The web server is available at https://huggingface.co/spaces/Xue-Jun/RT-Transformer.

## 1 Introduction

Metabolomics systematically identifies and quantifies all metabolites in a given organism or a biological sample (Idle and Gonzalez 2007). Metabolite annotation and identification is the main bottleneck in untargeted metabolomics (Neumann and Böcker 2010, van Der Hooft *et al.* 2016, Chong *et al.* 2018, Wishart *et al.* 2018). Liquid chromatography-mass spectrometry (LC-MS) has become the most widely used method for metabolite identification due to its enhanced resolution and excellent sensitivity (Bell *et al.* 2009, Gika *et al.* 2014). Generally, the liquid chromatography coupled to high resolution mass spectrometry (LC-HRMS) consists of information such as retention time (tR versus base ion/target ion Intensity) as well as many individual and centroid MS spectrum (m/z versus intensity). Therefore, various approaches (Allen *et al.* 2014, Ridder *et al.* 2014, Wang *et al.* 2014, Dührkop *et al.* 2015, Ruttkies *et al.* 2016, Wang *et al.* 2017, Djoumbou-Feunang *et al.* 2019, Dührkop *et al.* 2019, Ruttkies *et al.* 2019) have been developed to identify metabolites by searching against structural databases, such as PubChem (Kim *et al.* 2016) and ChemSpider (Hettne *et al.* 2010). Unfortunately, all of these approaches return multiple candidates with similar structures. To reduce the cost of the experiment, it is essential to filter out as many false candidates as possible. Previous studies have shown that retention times obtained by chromatographic separation can filter candidates with similar spectra but different retention times, further facilitating the identification of metabolites.

Experimental methods for obtaining retention times are costly; therefore, most datasets contain only a small fraction of known compounds. To predict compounds that lack experimental retention times, many researchers have developed different retention time prediction methods (Eugster *et al.* 2014, Cao *et al.* 2015, Stanstrup *et al.* 2015, Falchi *et al.* 2016, Bruderer *et al.* 2017, Amos *et al.* 2018, Aalizadeh *et al.* 2019, Pasin *et al.* 2021). Traditional machine learning approaches, such as multiple linear regression, random forest, support vector machine, and gradient boosting, are often used for retention time prediction (Aicheler *et al.* 2015, Wolfer *et al.* 2016, Bach *et al.* 2018, Bonini *et al.* 2020, Feng *et al.* 2021, Liapikos *et al.* 2022). For example, Bouwmeester *et al.* (2019) compared different ML methods for retention time prediction and found that an ensemble of several machine learning-based models performed best. Recently, small molecule retention time (SMRT) dataset (Domingo-Almenara *et al.* 2019) containing 80 038 molecules was released to the public, stimulating deep learning-based retention time prediction methods, such as DLM (Domingo-Almenara *et al.* 2019), DNNpwa (Ju *et al.* 2021), and 1D-CNN (Fedorova *et al.* 2022). More deep learning-based methods, such as GNN-RT (Yang *et al.* 2021), CPORT (Zaretckii *et al.* 2022), MPNN (Osipenko *et al.* 2022), and Blender (García *et al.* 2022), apply transfer learning (Weiss *et al.* 2016) to predict the retention times in specific chromatographic separation systems. These methods alleviate the limitation of small training data by pre-training the neural networks on SMRT and further reusing some parameters in the pre-trained networks. More specifically, GNN-RT (Yang *et al.* 2021) and MPNN (Osipenko *et al.* 2022) exploit graph neural networks (GNN) to learn effective molecular representations from the structures of small molecules, improving the transferability of the models.

These methods have been developed and have made significant progress in retention time prediction. However, retention times are determined by the combination of the metabolite with the chromatographic condition, so the retention time of the same metabolite can vary from different labs. Most current methods heavily rely on molecular fingerprints or descriptors while neglecting node and edge attributes in molecular structures, resulting in the inability to learn effective molecular representations. How to exploit and combine molecular fingerprints with structures to improve RT prediction is still an open issue. Third, recent methods, such as GNN-RT (Yang *et al.* 2021), attempt to use GNNs to embed a molecule structure as a fixed feature vector for RT prediction. However, GNNs over-emphasize proximity and tend to treat atoms and chemical bonds equally, making models preclude efficient global propagation and thus limiting the generality of the learned molecular representations. Graph attention networks (GAT) (Veličković *et al.* 2017), one of the most popular GNN architectures, use the attention mechanism (Bahdanau *et al.* 2014) to update the attributes of every node, allowing GAT to better exploit the graph structure, node information, and edge information to obtain the nodes’ representations in low-dimensional space. Insufficient availability of data poses a challenge for graph-based algorithms to effectively learn the required features. Hence, leveraging molecular fingerprints as Supplementary Information can potentially serve as an effective approach in addressing this limitation.

Based on these, we propose a new deep neural network model, RT-Transformer, combining GAT (Veličković *et al.* 2017) and 1D-Transformer, which is a module we developed based on the Transformer Encoder (Vaswani *et al.* 2017), to learn the effective molecular representations from molecular graphs and molecular fingerprints for RT prediction. We train the model using the SMRT dataset, freeze the feature extraction layer of the resulting model, and further fine-tune the model on other CM-specific datasets for prediction. The experimental results show that our model significantly outperforms the previous methods. In addition, the pre-trained model on SMRT dataset is evaluated with 41 external retention time datasets obtained from PredRet (Stanstrup *et al.* 2015).

## 2 Materials and methods

### 2.1 Preparation of the SMRT dataset

The SMRT dataset from the METLIN library was released by Domingo-Almenara *et al.* (2019). It provides RT data for 80 038 small molecules, including metabolites, natural products, and drug-like small compounds, all obtained using RP chromatography and HPLC-MS. Previous studies (Domingo-Almenara *et al.* 2019, Ju *et al.* 2021, Yang *et al.* 2021, Fedorova *et al.* 2022, Osipenko *et al.* 2022) omitted the new molecules and trained models with the molecules with retention periods longer than 300 s. To evaluate the models comprehensively, we pre-trained two models with retained molecules and all molecules in SMRT, respectively. The two-pertained models were then transferred to several datasets and compared with state-of-art models. The detailed information regarding the processing of molecular graph data and molecular fingerprint can be found in the Supplementary Information.

### 2.2 Overview of RT-Transformer

**Figure 1.**
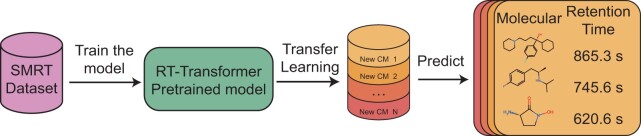
Procedure of RT prediction by RT-Transformer. In the initial phase, a model is pre-trained on the SMRT dataset. Subsequently, using transfer learning methodology, individuals have the capability to fine-tune the model using their own dataset. This process enables the acquisition of a retention time prediction model applicable to their specific chromatographic conditions.

RT-Transformer takes the resulting molecular graphs and Morgan fingerprints using InChl (International Chemical Identifier) as input, and extracts the features using a multi-head GAT and a stacked 1D-transformer, respectively. Next, the obtained features are fused and fed into a linear layer to produce a vector for RT prediction. An overview of RT-transformer is illustrated in Fig. 1.

### 2.3 ResGAT

Graph Attention Networks (GAT) were proposed by Veličković *et al.* (2017) to learn graph-structured data based on attention mechanism (Bahdanau *et al.* 2014). We devise a ResGAT block, consisting of a three-head GAT with a residual connection, to learn the embedding vectors of the molecular graphs. The ResGAT block uses a linear layer as an aggregation function instead of an addition or concatenation function to speed up the training process. This allows each node to aggregate information from all other nodes. Then, the node features are updated by adding the nonupdated features through skip connections. Finally, we apply layer normalization (Ba *et al.* 2016) to all node features.

$$\begin{array}{c} y=\frac{x-E[x]}{\sqrt{\text{Var}[x]+\epsilon }}*\gamma +\beta \end{array}$$

The mean and standard deviation are calculated over the last *D* dimensions, where *D* is the dimension of the inputs. γ and β are learnable affine transform parameters.

The input of this block is node features $h=\left\{\vec{h_{1}},\vec{h_{2}},\ldots ,\vec{h_{N}}\right\}$ and edge features $d=\left\{\vec{d_{1}},\vec{d_{2}},\ldots ,\vec{d_{M}}\right\}$, where $\vec{h_{i}}\in R^{F},\vec{d_{i}}\in R^{G}$, *N* and *M* are the numbers of nodes and edges, respectively; *F* and *G* are the dimensions of a node feature and an edge feature, respectively. For any two nodes, their features ${\vec{h}}_{i}$ and ${\vec{h}}_{j}$ and the features ${\vec{d}}_{ij}$ of the bond between them are transformed into a vector $e_{ij}$ and then the attention coefficient $a_{ij}$ between these two nodes is calculated as follows:

$$\begin{array}{c} \begin{array}{c} e_{ij}=\mathrm{Attention}\left(W{\vec{h}}_{i},W{\vec{h}}_{j},W{\vec{d}}_{ij}\right) \end{array}\\ \begin{array}{c} a_{ij}=\text{Softmax}\left(e_{ij}\right)=\frac{\;\text{exp}\;\left(e_{ij}\right)}{\sum_{k\in N_{i}}\;\text{exp}\;\left(e_{ik}\right)} \end{array} \end{array}$$

where W is a weight matrix; *Attention* is a function of attention mechanism. The specific implementation of this function, *Attention*, is as follows:

$$\begin{array}{c} a_{ij}=\frac{\;\text{exp}\;\left(\phi \left({\vec{\alpha }}^{⊤}\left[W{\vec{h}}_{i}\left\|W{\vec{h}}_{j}\right\|W{\vec{d}}_{ij}\right]\right)\right)}{\sum_{k\in N_{i}}\;\text{exp}\;\left(\phi \left({\vec{\alpha }}^{⊤}\left[W{\vec{h}}_{i}\left\|W{\vec{h}}_{k}\right\|W{\vec{d}}_{ik}\right]\right)\right)} \end{array}$$

where α is a 2N+M dimensional vector; || is the concatenation operation; ϕ is a LeakyReLU activation function as follows:

$$\begin{array}{c} \phi =\mathrm{LeakyReLU}(x)=\{\begin{array}{c} x,x>0\\ \lambda x,x\leq 0 \end{array} \end{array}$$

where λ is 0.0001. After getting the attention coefficient, we could calculate the final output features of every node (after potentially applying a nonlinearity).

The updated features of node *i* are as follows:

$$\begin{array}{c} {\vec{h}\prime }_{i}=\sigma \left(\sum_{j\in N_{i}}a_{ij}W{\vec{h}}_{j}\right) \end{array}$$

Because this block uses multi-head attention, we concentrate all vectors generated by all heads as follows:

$$\begin{array}{c} {\vec{h}\prime }_{i}=\text{concat}\left(\sigma \left(\sum_{j\in N_{i}}\alpha_{ij}^{k}W^{k}\vec{h_{j}}\right)\right) \end{array}$$

**Figure 2.**
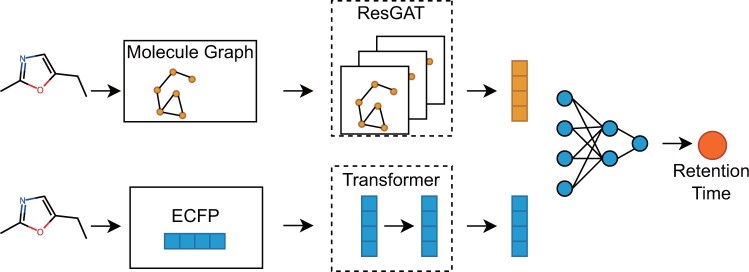
Structure of RT-Transformer. In the handling of molecular graph data, the ResGAT module is used, while the transformer module is utilized for fingerprint data processing. The outputs of these two modules are concatenated and fed through a multi-layer neural network to ultimately predict the retention time.

The detail of ResGAT is shown in Fig. 2.

### 2.4 1D-Transformer

The 1D-Transformer is based on Transformer Encoder architecture (Vaswani *et al.* 2017). Transformer, an encoder–decoder architecture, is proposed for machine translation tasks and has achieved state-of-art performance in many deep learning areas such as computer vision, and natural language processing. The 1D-Transformer is a combination of attention layers and feed-forward layers. The attention layer exploits the scaled-dot attention mechanism to capture the features most relevant to RTs, and takes three inputs, i.e. the keys K, the queries Q, and the values V. To make the attention layer more robust, we add a trainable matrix W and compute the attention as follows.

$$\begin{array}{c} \text{Attention}\left(Q,K,V\right)=\text{softmax}\left(\frac{QK^{⊤}}{\sqrt{D}}\right)VW \end{array}$$

The dot product of Q and K computes how closely the keys are aligned with the queries. If the query and key are aligned, their dot product will be big, and vice versa. Each key has a value vector multiplied by the softmax output, normalizing the dot products and emphasizing the most significant components. Q, K, and V are all the input vectors $\vec{f}\in R^{D}$, where *D* is the number of the input features. The feed-forward layers are composed of 2 linear layers. The detail of 1D-Transformer is shown in Fig. 2.

### 2.5 Output-block

Output-Block receives molecular graphs output by ResGATs and fingerprints proceed by 1D-Transformers. We used an addition function to readout the molecular graph, ignoring the features of bonds on the graph. Besides, the readout layer adopts a linear layer with a 512D input channel and a 512D output channel so that the features produced by ResGAT are suitable for feature fusion. Then, two linear layers are used to reduce the dimension of the fused features. The features extracted by ResGAT and 1D-Transformer have been resized to 512 dimensions by several linear layers. A 1024D vector concatenating these features goes through 3 linear layers and produces a vector for RTs prediction. All the linear layers are activated by Rectified Linear Unit function (ReLU).

### 2.6 RT-Transformer

RT-Transformer could be divided into three modules: ResGAT, 1D-Transformer, and Output-Block. A linear layer embeds the node features into 512 dimensions to get the higher dimension relationship. ResGAT receives molecular graphs as input. By stacking 9 ResGAT Blocks, every atom could get information from other atoms and chemical bonds. At the end of ResGATs, we use an addition function to readout all the atom features into the molecular graph features. 1D-Transformer receives a molecular fingerprint, a 2048D vector, as its input, and produces a 2048D vector as its output. We stack 12 1D-Transformer blocks to process the fingerprint feature. After processing these features, we send them to Output-Block to get the final prediction.

### 2.7 Transfer learning

We applied transfer learning to adapt our model to different chromatographic conditions. During the transfer learning, we fixed the weights of the ResGAT and the 1D-Transformer components, and initialized all the parameters in the Output-Block. We used the AdamW optimizer and trained the model for 300 epochs. The details of the training procedure are provided in the Supplementary Material.

## 3 Results and discussion

### 3.1 Evaluation of the RT-Transformer model

The SMRT dataset is a publicly accessible data collection that can be used to assess RT prediction models. Previous studies (Domingo-Almenara *et al.* 2019, Ju *et al.* 2021, Yang *et al.* 2021, Fedorova *et al.* 2022, Osipenko *et al.* 2022) have used SMRT retained molecules RTs as training data for their models. However, our research indicates that models trained with the complete SMRT dataset exhibit superior performance when transferred to other chromatographic methods (CMs) in some datasets. The performance improvement may benefit from the model’s ability to acquire additional features from the unretained molecules. Consequently, we have trained our model using both the complete SMRT dataset and a subset of the SMRT dataset that exclusively contains molecules with retention times >300 s. Specifically, when we utilized only the retained SMRT molecules to train our RT-Transformer, our model achieved a mean absolute error (MAE) of 27.30 s and a median absolute error (MedAE) of 12.46 s on the test set. By comparison, the MAE and MedAE errors of the model trained with all SMRT molecules are 33.41 and 13.05 s, respectively. A comparison of the accuracy of our RT-Transformer model with previous works is presented in Table 1. All data in this table are reproduced from the results reported in the original paper. In general, RT-Transformer is superior to the other models in all the performance metrics, including MAE, MRE, MedAE, MedRE, and *R*^2^. In the Supplementary Information, diverse experiments pertaining to the model, encompassing distinct parameters, along with details on the calculation of metrics presented in the table, are provided.

**Table 1.** Retention time prediction metrics of different models.^a^

Methods	MAE(s)	MRE (%)	MedAE(s)	MedRE (%)	*R* ^2^
GNN-RT (Yang et al. 2021)	39	5	24		0.85
1D-CNN (Fedorova *et al.* 2022)	34.7	4.3	18.7	2.4	
Blender (García *et al.* 2022)	34		17.2		
CPORT (Zaretckii *et al.* 2022)	44	5.5	26	3.4	
MPNN (Osipenko *et al.* 2022)	31.5	4	16		0.879
RT-Transformer	**27.30**	**3.42**	**12.46**	**1.58**	**0.88**

a The values in the table are extracted from the original research paper, with missing values denoting instances where the original paper did not provide the information. Values in boldface indicate the best-performing metrics among the compared models.

### 3.2 RT-Transformer transfer learning on other chromatographic systems

Currently, specific chromatographic methods usually suffer from insufficient training data, and different chromatographic methods (CM) may result in different RTs for the same metabolite. To address the issue of model overfitting on small datasets, transfer learning becomes an excellent alternative.

To evaluate the efficacy of the transfer learning approach, we utilized 41 datasets obtained from PredRet (Stanstrup *et al.* 2015), which were generated by diverse chromatographic methods (CMs) and contributed by researchers from independent laboratories.

We pre-trained the two RT-transformer models using retained molecules and all molecules in SMRT, respectively. We then froze the parameters of ResGATs and 1D-transformer modules in the pre-trained models, and fine-tuned the parameters of the Output-Block using target data to obtain RT prediction models for various CMs. A total of 41 datasets containing over 80 instances each were selected from the PredRet dataset collection. Transfer learning experiments were then conducted on these datasets by applying models pre-trained on large-scale datasets. The results of these transfer learning, including the means and standard deviations of MAE, MedAE, MRE, MedRE, and *R*^2^ determined via 10-fold cross-validation, are presented in Supplementary Tables S1–S4. To ensure the data partitioning is not affected by the random seed selection, we performed 10-fold cross validation on the dataset using five different random number generator seeds—1234, 12 345, 123 456, 1 234 567, and 12 345 678, respectively.

**Figure 3.**
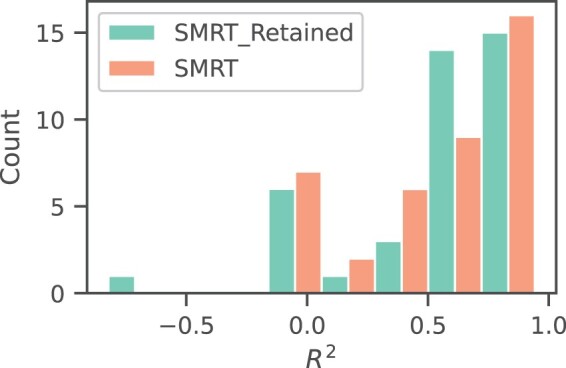
Distribution of transfer learning performance (measured by *R*^2^ score) for RT-Transformer trained by different training set across 41 datasets.

The distribution of *R*^2^ scores after transferring the methods trained on SMRT and SMRT Retained to 41 external datasets is shown in Fig. 3. The results presented above were obtained by averaging over these five trials. Due to the lack of detailed information on the dataset provided by the PredRet platform, we are unable to determine why the model cannot be migrated to some datasets. As evidenced in the figure, the model trained on the complete SMRT dataset exhibits higher stability, with no instances of *R*^2^ scores below −0.5. This enhanced stability may stem from the model learning additional molecular features from unreserved molecules, rendering it more adaptable. Furthermore, when *R*^2^ exceeds 0.75, the fully trained model outperforms the model trained solely on reserved molecules.

Part of the reason may be that the data was uploaded by third-party users, resulting in low data quality. Furthermore, since these well-established datasets have not been used for RT prediction, we use them as benchmarks and compare the performance of our model with other models established with previously tested datasets. Table 2 shows the transfer performance of our model and other state-of-the-art models on these benchmarks. As shown in Table 2, our model outperformed the state-of-the-art models on most evaluated metrics and datasets.

**Table 2.** MAEs of different models on multiple datasets.^a^

Model	RIKEN Retip	FEM_long	Eawag_XBridgeC18	LIFE_new	LIFE_old
GNN-RT (Yang *et al.* 2021)	—	235.01	112.78	29.38	17.1
1D-CNN (Fedorova *et al.* 2022)	32.4	—	—	23.6	15.5
MPNN (Osipenko *et al.* 2022)	38.2	204.6	80.9	22.1	16.9
RT-Transformer(SMRT_Retained)	37.16	184.04	73.34	**20.36**	**11.57**
RT-Transformer(SMRT)	**32.10**	**176.53**	**69.8**	22.12	13.09

a The values in the table are extracted from the original research paper, with missing values denoting instances where the original paper did not provide the information. Values in boldface indicate the best-performing metrics among the compared models.

### 3.3 Application and evaluation of RT prediction in compound annotation

Currently, metabolite identification methods based on MS^2^ spectra always propose different candidate motifs, resulting in a large number of false positive compounds. Especially when the molecules are structurally similar, it may be difficult to identify them. RTs contain information that is orthogonal to the mass spectra. It helps filter candidates, even if they have similar structures. The histogram in Supplementary Fig. S1 shows prediction errors of 7790 molecules in the test set. As can be seen from the figure, prediction errors can be regarded as a normal distribution. More specifically, there are 6306 molecules (89.84%) with an absolute error of <60 s and 6726 molecules (95.82%) with an absolute error of <120 s in the RT prediction. Using ±2 SD (standard deviation of RT prediction errors in the validation set is 52.80 s) as the filter threshold (Yang *et al.* 2021), the false negative rate decreases to 4.93%. Also, the threshold can be adjusted to filter more false positive molecules or minimize false negative molecules.

**Figure 4.**
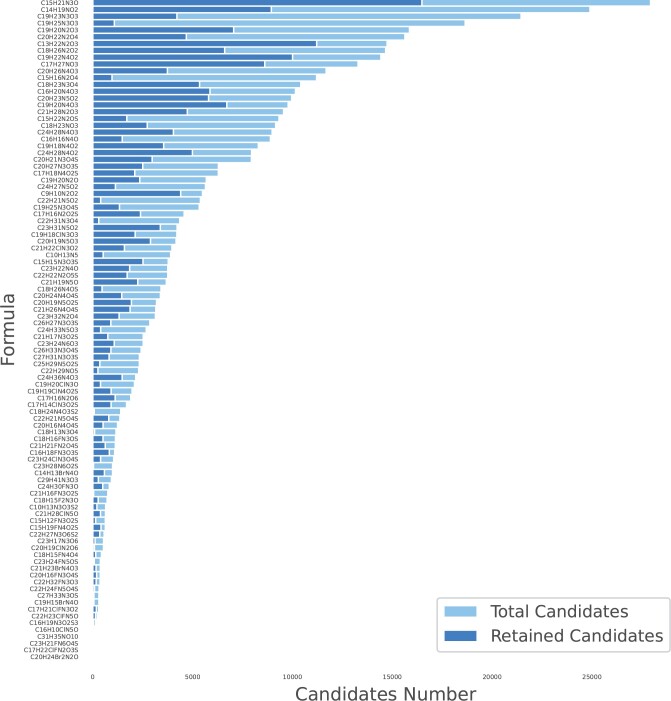
The Filtering Ability of RT-Transformer Filter. The isomeric count for 100 molecules, both before and after filtration based on predicted retention time (RT), indicates a strong discriminatory capability of RT-based filtering for distinguishing isomers. “Total Candidates” represents the total number of isomers for the molecular formula, while “Retained Candidates”represents the number of isomers remaining after filtering based on the predicted retention time.

We randomly selected 100 molecules from the test set of SMRT. And the visualization in Supplementary Fig. S2 presents that these molecules were distributed uniformly in the SMRT dataset. Then we searched PubChem with the molecular formula to get all the isomers as their candidates. All the candidate molecules were filtered by RT-Transformer filter. The number of selected candidates and the number of candidates filtered by RT-Transformer are shown in Fig. 4. The detail is shown in Supplementary Table S5. It can be seen that the means of the filter rate in 100 test molecules were 59.35%. This result showed the ability of the RT-Transformer filter to filter out false positive molecules.

In addition, our model provides a filter for filtering out more isomers in nontargeted LC-MS workflow. We search isomers of all molecules from PubChem. To access the capacity of RT-Transformer in the compound annotation, we generated receiver operating characteristic (ROC) curves based on SMRT (test set) and SMRT_Retained (retained molecules test set), as shown in Supplementary Figs S4 and S5, respectively. The resulting curves were then utilized to determine the optimal threshold and filter out candidate compounds based on these values. The best threshold for SMRT and SMRT_Retained is 5.7% and 4.3%, and eliminating 80.89% and 85.42% false identity, respectively. To further clarify our filter’s effectiveness, we generated the boxplot with the *x*-axis representing the filter’s effectiveness and the *y*-axis representing the number of molecules. The boxplot showed a significant increase in the number of molecules filtered out, indicating a notable improvement in the filter’s performance. For SMRT (test set) and SMRT_Retained (retained molecules test set), the boxplot is illustrated in Supplementary Figs S4 and S5. Overall, these results indicate that RT-transformer filters can effectively filter false positive molecules with RT prediction.

In order to simulate the performance of this model in the real-world compound identification process, we acquired Metabobase dataset from MoNa. Subsequently, we used MS-Finder to generate candidate compounds. Utilizing the RT-Transformer, we conducted transfer learning to train a retention time prediction model tailored to the current dataset. The model was then subjected to 10-fold cross-validation for retention time prediction. In MS-Finder, a total of 93 compounds were successfully identified as correct candidates, among which 10 compounds were erroneously filtered out by the RT-Transformer. Following the filtration process, for the compounds with correct annotations falling outside the top 10 rankings, there was an average improvement of 11 positions.

This indicates that the application of the RT-Transformer yields significant efficacy in filtering out false positives. Across all spectra, this model demonstrates the capacity to filter out 52.64% of candidate compounds, thereby substantially enhancing the effectiveness of nontargeted identification.

## 4 Conclusion

The retention time prediction in liquid chromatography is increasingly essential to identify small molecules, providing valuable orthogonal information to tandem mass spectra. In this study, we present a robust RT prediction model, RT-Transformer, designed to aid in identifying small molecules. The model exhibits excellent scalability across different chromatographic methods, and its performance was validated on both the SMRT dataset and 41 datasets obtained using various chromatographic methods. Our results indicate that RT-Transformer outperforms state-of-the-art models when trained on the SMRT dataset. By leveraging transfer learning, our model can accurately predict RT values in any chromatographic method and demonstrate superior performance to other RT prediction models. Our findings demonstrate that RT-Transformer can filter isomeric candidates based on their predicted RT values, thereby facilitating molecular identification. Furthermore, we have made the source code and pretrained-model of RT-Transformer publicly available, enabling researchers to apply this model to their datasets via transfer learning and improve the accuracy and efficiency of their chemical analyses.

## Supplementary Material

btae084_Supplementary_Data
