# Supplementary material for: RT-Transformer: retention time prediction for metabolite annotation to assist in metabolite identification
Source: Bioinformatics. 2024 Feb 24;40(3):btae084. doi: 10.1093/bioinformatics/btae084 (PMC10914443; doi:10.1093/bioinformatics/btae084)
Supplement: btae084_Supplementary_Data [file btae084_supplementary_data.pdf]

Supplementary Information for

RT-TRANSFORMER: RETENTION TIME PREDICTION FOR METABOLITE ANNOTATION TO ASSIST IN METABOLITE IDENTIFICATION.

Jun Xue[1,2], Bingyi Wang[3,4],Hongchao Ji[2] and Weihua Li[1,*]

[1]School of Information Science and Engineering, Yunnan University, Kunming 650500, China

2Shenzhen Branch, Guangdong Laboratory for Lingnan Modern Agriculture, Genome Analysis Laboratory of the Ministry of Agriculture and Rural Affairs, Agricultural Genomics Institute at Shenzhen, Chinese Academy of Agricultural Sciences, Buxi

[3]Faculty of Drug Control, Yunnan Police College, Kunming 650223, China

[4]Key Laboratory of Smart Drugs Control(Yunnan Police College), Ministry of Education, Yunnan Police College, Kunming 650223, China

Corresponding Author

*Weihua Li, Mailing address: South Section, East Outer Ring Road, Chenggong District, Kunming, Yunnan province, People's Republic of China; E-mail: liweihua@ynu.edu.cn

Evaluation Metrics:

$$\mathrm{MAE} = \frac{1}{N}\sum_{i}^{N}\left|y_i - \hat{y}_i\right|$$

$$\mathrm{MRE} = \frac{1}{n}\sum_{i=1}^{n}\frac{\left|y_i - \hat{y}_i\right|}{|\, y_i \,|}$$

$$\mathrm{MedAE} = \mathrm{median}(|\, y_i - \hat{y}_i \,|)$$

$$\mathrm{MedRE} = \mathrm{median}(\frac{\left|y_i - \hat{y}_i\right|}{|\, y_i \,|})$$

$$R^2 = 1 - \frac{\sum_{i=1}^{n}(y_i - y_i)^2}{\sum_{i=1}^{n}\left(y_i - \bar{y}\right)^2}$$

Activate Function:

$$\mathrm{ReLU(x)} = \begin{cases} \mathrm{x, x > 0} \\ \mathrm{0, x \le 0} \end{cases}$$

Training Setups:

The SMRT dataset is randomly divided into non-overlapping training, validation, and test sets of 80%, 10%, and 10%, respectively. During training, the model seeks to minimize L1 loss in the validation set for a maximum of 300 epochs. The RT-transformer is trained through backpropagation and optimized using AdamW with an initial learning rate of 0.0001 that decays by a factor of 0.1 every 50 epochs. The batch size is set to 64. In the transfer learning phase, the model adopts an initial learning rate of 0.001 that decreases by a factor of 0.1 per 30 epochs, and the batch size is set to 8. The model trains for 130 epochs using the AdamW optimizer.

Molecular Graph Data

We represent molecules as graphs, with the nodes and edges representing the atoms and chemical bonds in the molecule, respectively. Each node is associated with several attributes, such as chirality, relative atomic mass, degree, formal charge, orbital hybrid mode, valence, radical electrons, whether or not it is on a ring. Each edge is assigned some attributes, including the type of the bond, whether it is a ring, aromatic, or conjugated.We transform all the attributes mentioned above into one-hot vectors and concatenate them with the value attribute. Besides, for each chemical bond in the molecular graphs, we convert it into a bi-directional edge.In this paper, we use Python package RDKit(www.rdkit.org) to generate molecular graphs with 34 features per node and 5 features per bond. The input of 1D-Transformer is the Extended-Connectivity Fingerprints (ECFP) containing 2048 bits ,which is calculated by RDKit.

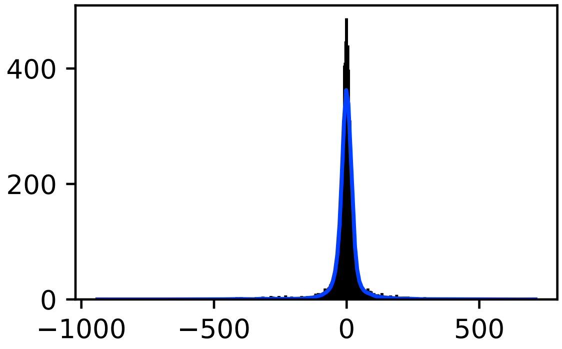

Figure S1 Error Distribution of SMRT

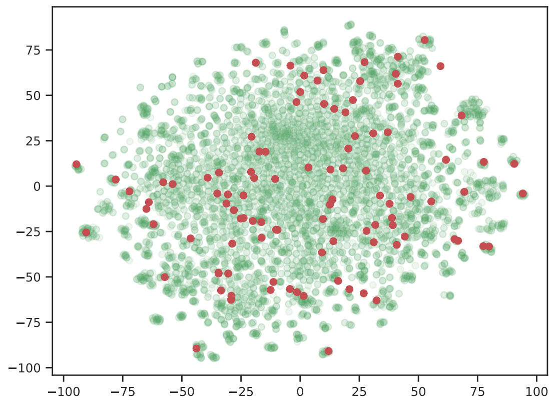

Figure S2 Distributions of 100 randomly selected molecules from the test set in SMRT

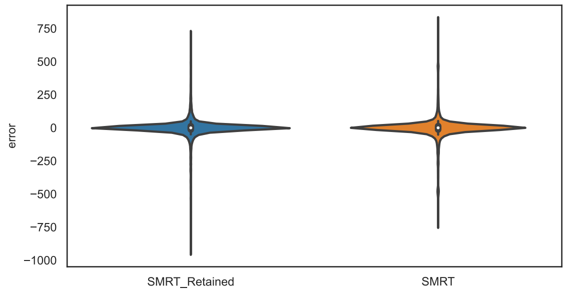

Figure S3 Error Distribution of SMRT and SMRT_Retained

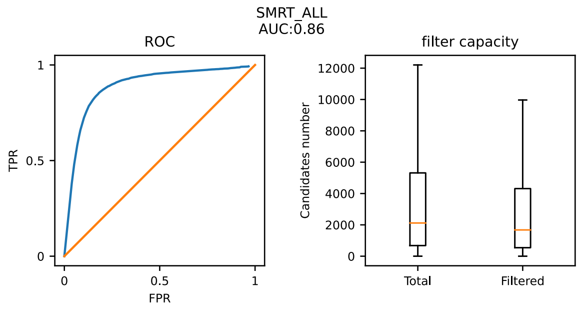

Figure S4 ROC curves and eliminated false identities of SMRT

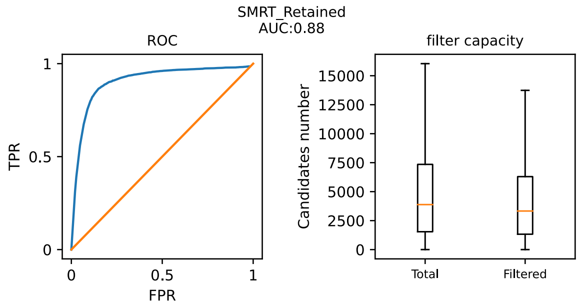

Figure S5 ROC curves and eliminated false identities of SMRT_Retained

Table S1 Means of 10-fold Cross-Validation transfer results on 41 CMs by the model trained with retained molecules

dataset	mae(s)	mre(%)	medAE(s)	medRE(%)	R2
Acquity HSST3-RP-60mn	328.73	49.1	238.73	19.12	0.62
AjsTestF	77.57	41.21	50.11	25.6	0.58
AjsUoB	61.25	51.01	32.91	26.6	0.68
BDD_C18	52.07	46.67	18.88	13.58	0.78
BfG_NTS_RP1	78.84	20.52	53.09	10.54	0.72
CBM_Test_A	150.65	41.93	131.5	34.07	-0.05
CBM_Test_A_	57.63	20.2	41.33	13.3	-0.15
CBM_Test_B	137.66	46.14	112.38	37.94	-0.04
CBM_Test_E	75.47	26.3	58.15	19.72	-0.01
CBM_Test_F	23.26	3.39	17.88	2.58	-0.82
CBM_Test_G	133.39	45.91	103.12	23.17	0.35
CS1	32.95	10.31	21.86	3.65	0.88
Cao_HILIC	101.74	64.94	52.28	36.21	0.48
Eawag_XBridgeC18	73.34	27.43	58.4	13.41	0.82
FEM_long	184.04	36.85	122.45	11.8	0.93
FEM_orbitrap_plasma	50.29	18.63	35.87	8.77	0.94
HILIC_BDD_2	148.64	47.83	91.77	17.93	0.54
HILIC_tip	80.51	42.63	50.79	26.49	0.51
IJM_TEST	92.66	51.26	36.84	11.3	0.27
IPB_Halle	48.75	38.98	29.04	20.61	0.64
KI_GIAR_zic_HILIC_pH2_7	97.36	43.87	80.22	23.03	0.51
LIFE_new	20.36	26.61	11.82	12.53	0.87
LIFE_old	11.57	16.04	8.27	9.31	0.91
MTBLS87	72.54	12.3	51.62	7.88	0.65
MTBLS_36	61.16	38.45	29.26	18.79	-0.01
Mceachran HPLC	50.54	26.08	42.32	16.19	0.7
Meister zic-pHILIC pH9.3	71.23	35.49	54.24	24.1	0.51
RIKEN	45.29	81.57	17.25	30.79	0.62
RPFDAMM	23.11	8.2	14.37	4.59	0.84
RPLC_zorbax150_JH	32.94	30.41	20.43	20.06	0.6
RPMMFDA	33.48	18.14	22.28	7.01	0.79
SNU_RIKEN_POS	37.39	15.12	28.6	10.83	0.8
SNU_RP_indole_annotation	34.34	16.31	18.94	7.53	0.94
SNU_RP_indole_order	36.89	17.47	24.88	8.65	0.83
SNU_organoid	95.81	43.91	60.87	11.99	0.87
UFZ_Phenomenex	128.79	21.63	81.19	6.18	0.64
UniToyama_Atlantis	44.8	5.12	27.5	3.05	0.9
Waters ACQUITY UPLC with Synapt G1 Q-TOF	135.62	48.76	97.53	31.2	0.29
Waters STA Forensic	132.33	54.71	110.49	32.78	-0.03
cecum_JS	36.89	17.47	24.88	8.65	0.83

Table S2 Standard deviation of 10-fold Cross-Validation transfer results on 41 CMs by the model trained with retained molecules

dataset	mae	mre	medAE	medRE	R2
Acquity HSST3-RP-60mn	87.15	39.14	90.71	8.17	0.18
AjsTestF	8.6	6.97	8.06	3.69	0.11
AjsUoB	6.64	6.53	7.39	5.04	0.07
BDD_C18	9.82	14.37	8.01	3.07	0.09
BfG_NTS_RP1	8.6	4.04	7.32	1.43	0.07
CBM_Test_A	10.39	4.09	18.57	3.27	0.05
CBM_Test_A_	23.79	8.72	26.84	8.05	0.3
CBM_Test_B	13.94	5.94	19.29	6.56	0.07
CBM_Test_E	15.3	5.64	15.91	5.56	0.13
CBM_Test_F	8.38	1.29	9.77	1.4	1.16
CBM_Test_G	43.49	26.09	45.35	15.43	0.39
CS1	11.44	12.14	7.96	1.22	0.07
Cao_HILIC	13.24	13.31	14.95	6.86	0.09
Eawag_XBridgeC18	8.19	8.26	11.57	2.81	0.05
FEM_long	40.01	17.49	37.76	3.88	0.04
FEM_orbitrap_plasma	20.21	13.51	19.84	4.83	0.05
HILIC_BDD_2	22.73	12.87	26.37	5.36	0.13
HILIC_tip	8.35	5.83	7.96	3.62	0.1
IJM_TEST	26.07	18.45	11.9	3.7	0.31
IPB_Halle	14.07	13.26	14.93	9.27	0.28
KI_GIAR_zic_HILIC_pH2_7	10.14	7.28	13.15	4.96	0.11
LIFE_new	6.39	11.79	5.77	5.84	0.08
LIFE_old	2.61	4.7	2.51	3.23	0.08
MTBLS87	27.52	5.9	28.99	3.74	0.29
MTBLS_36	35.53	22.21	25.23	8.55	1.16
Mceachran HPLC	12.39	7.49	11.64	5.31	0.21
Meister zic-pHILIC pH9.3	14.83	8.32	16.65	6.5	0.18
RIKEN	16.32	25.34	5.39	9.64	0.28
RPFDAMM	9.54	4.65	8.75	2.95	0.14
RPLC_zorbax150_JH	7.24	7.78	6.26	6.07	0.25
RPMMFDA	3.24	3.79	2.15	0.78	0.06
SNU_RIKEN_POS	3.42	1.71	3.31	1.37	0.04
SNU_RP_indole_annotation	14.15	9.37	11.09	3.51	0.06
SNU_RP_indole_order	18.78	13.29	16.19	5.5	0.37
SNU_organoid	47.68	38.69	37.89	4.47	0.13
UFZ_Phenomenex	30.13	12.7	18.94	1.46	0.11
UniToyama_Atlantis	16.1	1.99	15.09	1.69	0.08
Waters ACQUITY UPLC with Synapt G1 Q-TOF	17.44	8.49	15.37	4.68	0.22
Waters STA Forensic	19.06	15.5	25.62	8.26	0.07
cecum_JS	18.78	13.29	16.19	5.5	0.37

Table S3 Means of 10-fold Cross-Validation transfer results on 41 CMs by the model trained with all molecules

dataset	mae	mre	medAE	medRE	R2
Acquity HSST3-RP-60mn	311.33	32.39	225.11	17.42	0.65
AjsTestF	73.95	38.07	45.75	23.49	0.6
AjsUoB	51.92	45.91	28.19	21.17	0.77
BDD_C18	47.95	49.22	20.36	12.53	0.78
BfG_NTS_RP1	76.16	18.76	49.77	10.13	0.72
CBM_Test_A	158.05	43.76	139.76	35.29	-0.07
CBM_Test_A_	76.62	25.92	59.44	19.15	-0.1
CBM_Test_B	141.89	47.65	115.75	37.55	-0.07
CBM_Test_E	85.32	28.26	67.59	21.78	0.03
CBM_Test_F	53.58	11.38	21.13	3.1	-0.07
CBM_Test_G	174.07	61.69	125.25	29.07	-0.02
CS1	36.63	20.73	16.9	2.89	0.85
Cao_HILIC	105.88	60.98	52.18	34.46	0.5
Eawag_XBridgeC18	69.8	22.65	48.04	12.52	0.81
FEM_long	176.53	47.24	106.03	10.46	0.91
FEM_orbitrap_plasma	68.28	22.54	39.34	9.14	0.88
HILIC_BDD_2	116.6	38.25	57.87	11.87	0.66
HILIC_tip	73.69	38.1	44.9	24.07	0.58
IJM_TEST	104.98	44.6	31.15	9.8	0.26
IPB_Halle	27.97	24.21	15.53	10.91	0.86
KI_GIAR_zic_HILIC_pH2_7	81.02	34.77	59.41	16.98	0.64
LIFE_new	22.12	30.29	9.87	9.93	0.81
LIFE_old	13.09	17.31	7.65	8.94	0.86
MTBLS87	138.18	22.27	86.9	13.56	0.33
MTBLS_36	93.06	42.57	38.43	20.72	0.18
Mceachran HPLC	84.21	33.01	47.58	18.3	0.32
Meister zic-pHILIC pH9.3	80.18	34.06	50.63	23.13	0.42
RIKEN	50.7	61.01	11.51	27.15	0.52
RPFDAMM	33.74	26.27	18.51	6.04	0.76
RPLC_zorbax150_JH	47.36	38.89	24.5	19.77	0.35
RPMMFDA	30.9	14.69	20.94	6.28	0.82
SNU_RIKEN_POS	35.85	13.86	25.56	9.64	0.8
SNU_RP_indole_annotation	42.59	17.97	17.68	7.41	0.88
SNU_RP_indole_order	46.09	19.16	21.99	8.47	0.87
SNU_organoid	151.22	51.93	62.88	16.57	0.59
UFZ_Phenomenex	105.13	13.78	67.53	5.3	0.79
UniToyama_Atlantis	95.3	13.32	55	5.99	0.67
Waters ACQUITY UPLC with Synapt G1 Q-TOF	123.82	48.71	87.91	26.85	0.43
Waters STA Forensic	149.06	56.21	125.59	36.79	-0.08
cecum_JS	46.14	19.22	22.04	8.51	0.87

Table S4 Standard deviation of 10-fold Cross-Validation transfer results on 41 CMs by the model trained with all molecules

dataset	mae	mre	medAE	medRE	R2
Acquity HSST3-RP-60mn	62.22	9.86	83.4	6.5	0.17
AjsTestF	7.84	6.12	9.13	3.65	0.09
AjsUoB	5.77	8.59	4.59	5.31	0.06
BDD_C18	11.99	18.69	9.87	3.7	0.15
BfG_NTS_RP1	8.77	3.69	5.68	1.29	0.11
CBM_Test_A	11.73	4.84	20.14	3.63	0.06
CBM_Test_A_	20.96	8.47	33.88	10.37	0.29
CBM_Test_B	15.08	7.42	19.69	6.5	0.1
CBM_Test_E	14.46	5.57	19.47	5.3	0.13
CBM_Test_F	31.98	8.79	13.37	2.05	0.63
CBM_Test_G	34.52	20.47	51.31	17.34	0.36
CS1	15.26	21.12	8.44	1.36	0.12
Cao_HILIC	14.4	13.02	15.08	6.22	0.08
Eawag_XBridgeC18	13.61	6.51	10.23	3.17	0.11
FEM_long	42.36	31.66	30.43	3.03	0.09
FEM_orbitrap_plasma	33.48	15.63	26.15	4.29	0.15
HILIC_BDD_2	18.95	8.51	17.75	3.12	0.11
HILIC_tip	7.13	4.79	7.26	3.97	0.09
IJM_TEST	27.62	19.84	11.04	3.68	0.27
IPB_Halle	10.84	13.17	10.88	6.89	0.18
KI_GIAR_zic_HILIC_pH2_7	9.94	5.99	12.09	2.6	0.12
LIFE_new	6.04	11.86	4.32	4.26	0.12
LIFE_old	4.03	6.31	3	2.86	0.15
MTBLS87	51.15	7.63	46.76	7.23	0.38
MTBLS_36	39.46	18.71	38.86	12.47	0.73
Mceachran HPLC	34.19	11.64	19.06	6.47	0.33
Meister zic-pHILIC pH9.3	20.68	8.58	17.66	6.38	0.23
RIKEN	15.08	22.46	7.18	15.06	0.28
RPFDAMM	19.4	42.98	9	2.61	0.31
RPLC_zorbax150_JH	20.27	13.37	11.29	6.21	0.48
RPMMFDA	3.01	2.64	2.36	0.65	0.07
SNU_RIKEN_POS	3.95	1.51	3.84	1.6	0.04
SNU_RP_indole_annotation	18.66	12.09	11.4	3.75	0.11
SNU_RP_indole_order	24.32	17.37	18.13	4.35	0.15
SNU_organoid	87.31	29.18	44.94	13.66	0.46
UFZ_Phenomenex	15.33	5.01	15.04	1.23	0.09
UniToyama_Atlantis	45.24	10.81	41.01	4.24	0.24
Waters ACQUITY UPLC with Synapt G1 Q-TOF	14.87	9	17.92	5.89	0.15
Waters STA Forensic	22.29	15.54	30.47	7.96	0.1

cecum_JS	24.3	17.34	18.13	4.32	0.15

Table S5 The results of RT-Transformer trained with retained molecules of SMRT in ranking capability

Formula	experimental	Num of candidates searched from PubChem	Num of candidates filtered by RT-Transformer	Rate of candidates filtered by RT-Transformer	Candidate is filtered out
C16H16N4O	796.30	8896	6230	70.03	TRUE
C27H33N3OS	855.80	307	253	82.41	FALSE
C15H21N3O	518.30	27937	11452	40.99	TRUE
C23H28N6O2S	1108.20	992	957	96.47	TRUE
C19H23N3O3	888.90	21452	17240	80.37	TRUE
C21H23BrN4O3	621.40	375	212	56.53	TRUE
C22H22N2O5S	802.50	3753	2032	54.14	TRUE
C19H20N2O3	869.60	15864	8810	55.53	TRUE
C16H20N4O3	709.00	10154	4283	42.18	TRUE
C21H19N5O	673.80	3683	1432	38.88	TRUE
C17H14ClN3O2S	903.00	1691	774	45.77	TRUE
C23H21FN6O4S	782.70	101	32	31.68	TRUE
C20H19N5O2S	737.80	3192	1259	39.44	TRUE
C23H17N3O6	1198.20	541	426	78.74	TRUE
C23H32N2O4	764.50	3137	1820	58.02	TRUE
C19H19ClN4O2S	891.50	1966	1053	53.56	TRUE
C18H26N4OS	914.50	3414	2949	86.38	TRUE
C24H27N5O2	858.60	5646	4515	79.97	TRUE
C18H15F2N3O	647.60	715	449	62.80	TRUE
C22H21N5O2	1083.70	5390	4998	92.73	TRUE
C14H19NO2	821.00	24904	15960	64.09	TRUE
C24H28N4O2	692.70	7952	2961	37.24	TRUE
C19H15BrN4O	1146.60	305	250	81.97	TRUE
C18H13N3O4	1147.90	1163	1074	92.35	TRUE
C17H27NO3	685.70	13286	4673	35.17	FALSE
C20H24N4O4S	791.70	3384	1936	57.21	TRUE
C22H27N3O6S2	747.30	569	222	39.02	TRUE
C10H13N5	726.40	3899	3379	86.66	TRUE
C21H26N4O4S	617.40	3155	1287	40.79	TRUE
C17H16N2O2S	800.20	4575	2188	47.83	TRUE
C13H22N2O3	561.20	14740	3529	23.94	TRUE
C15H15N3O3S	728.00	3777	1264	33.47	TRUE
C24H36N4O3	627.00	2151	682	31.71	TRUE
C16H19N3O2S3	972.60	148	106	71.62	TRUE
C22H31N3O4	1062.60	4355	4059	93.20	TRUE
C18H26N2O2	745.50	14671	8068	54.99	TRUE
C17H22ClFN2O3S	885.20	69	40	57.97	TRUE
C25H29N5O2S	1058.80	2331	1980	84.94	TRUE

C17H18N4O2S	838.00	6287	4188	66.61	TRUE
C16H10ClN5O	796.70	135	65	48.15	TRUE
C19H22N4O2	604.30	14431	4423	30.65	TRUE
C20H21N3O4S	918.00	7945	4971	62.57	TRUE
C23H22N4O	673.40	3755	1908	50.81	TRUE
C22H21N5O4S	761.00	1358	560	41.24	TRUE
C15H22N2OS	933.60	9324	7621	81.74	TRUE
C20H16FN3O4S	924.00	373	181	48.53	TRUE
C19H18ClN3O3	816.10	4212	2089	49.60	TRUE
C22H23ClFN5O	723.10	235	98	41.70	FALSE
C26H33N3O4S	1017.50	2420	1513	62.52	TRUE
C24H28N4O3	852.00	8984	6471	72.03	TRUE
C20H23N5O2	697.10	9960	4160	41.77	TRUE
C21H21FN2O4S	872.00	1128	504	44.68	TRUE
C18H23NO3	621.60	9160	6433	70.23	TRUE
C23H24ClN3O4S	795.70	1046	657	62.81	TRUE
C19H20ClN3O	1058.00	2089	1706	81.67	TRUE
C19H25N3O4S	928.60	5333	3998	74.97	FALSE
C18H24N4O3S2	1104.50	1400	1328	94.86	TRUE
C22H29NO5	1246.60	2295	2040	88.89	TRUE
C17H21ClFN3O2	705.40	298	122	40.94	TRUE
C16H18FN3O3S	720.70	1096	302	27.55	TRUE
C15H12FN3O2S	976.10	627	486	77.51	TRUE
C20H16N4O4S	681.20	1242	728	58.62	TRUE
C15H16N2O4	975.60	11207	10246	91.43	TRUE
C20H22N2O4	603.40	15634	10948	70.03	FALSE
C20H27N3O3S	776.90	6293	3793	60.27	TRUE
C23H24N6O3	565.60	2525	1453	57.54	TRUE
C21H16FN3O2S	1285.30	751	708	94.27	TRUE
C22H32FN3O3	724.20	363	195	53.72	FALSE
C19H18N4O2	799.10	8289	4742	57.21	TRUE
C20H24Br2N2O	906.20	42	29	69.05	TRUE
C21H28ClN5O	585.00	636	252	39.62	TRUE
C21H22ClN3O2	811.90	3962	2381	60.10	TRUE
C24H30FN3O	642.20	828	329	39.73	TRUE
C29H41N3O3	924.40	933	658	70.53	TRUE
C21H28N2O3	653.70	9554	4821	50.46	TRUE
C21H17N3O2S	745.80	2527	1776	70.28	TRUE
C10H13N3O3S2	738.10	645	438	67.91	TRUE
C18H15FN4O4	907.30	434	287	66.13	TRUE
C23H31N5O2	646.40	4223	842	19.94	TRUE
C31H35NO10	721.20	107	62	57.94	TRUE
C18H23N3O4	750.00	10416	5066	48.64	TRUE
C19H25N3O3	1021.00	18650	17574	94.23	TRUE
C20H19N5O3	649.60	4171	1280	30.69	TRUE
C20H26N4O3	783.00	11688	7952	68.04	TRUE

C24H33N5O3	873.90	2678	2283	85.25	TRUE
C20H19ClN2O6	1143.40	536	454	84.70	FALSE
C19H20N2O	879.00	5684	3335	58.67	TRUE
C16H18FN3O3S	685.30	1096	233	21.26	TRUE
C15H19FN4O2S	736.00	625	215	34.40	TRUE
C14H13BrN4O	684.80	989	409	41.35	TRUE
C9H10N2O2	655.40	5495	1095	19.93	TRUE
C24H28N4O3	656.60	8984	3419	38.06	TRUE
C16H16N4O	1025.20	8896	8615	96.84	TRUE
C19H20N4O3	642.00	9785	3070	31.37	FALSE
C23H24FN5OS	1108.70	377	307	81.43	FALSE
C22H24FN5O4S	922.20	314	231	73.57	TRUE
C27H31N3O3S	674.10	2334	1520	65.12	TRUE
C17H16N2O6	777.50	1900	782	41.16	TRUE
C26H27N3O3S	830.90	2862	1959	68.45	TRUE
C18H16FN3OS	783.60	1136	627	55.19	TRUE

Table S6 The Features of Node and Edge in Molecular Graph

Node Feature	Edge Feature
IsAromatic	BondType
Symbol	BondDir
ChiralTag	IsInRing
AtomicNum	IsAromatic
Degree	IsConjugated
Mass	
TotalDegree	
FormalCharge	
Hybridization	
ExplicitValence	
ImplicitValence	
TotalValence	
AtomMapNum	
Isotope	
NumRadicalElectrons	
IsInRing	

Table S7 The MAEs of Learning Rate and Batch Size on the Mean Absolute Error of a Regression Model

lr / batch size	0,00001	0,00005	0,0001	0,0005
32	38.14	35,48	38,73	59,88
64	35,31	33,34	32,33	49,46
128	34,99	35,56	34,63	36,01
256	36,74	38,42	41,87	35,16

Table S8 MAEs of Different layer of ResGAT.

Layer num	MAE
1	82.58
2	73.64
3	54.93
4	787.77
5	787.55
6	42.48
7	41.65
8	41.54
9	40.87
10	40.62

Table S9 MAEs of Different layer of 1D-Transformer.

Layer num	MAE
1	170.60
2	149.35
3	141.03
4	164.43
5	151.58
6	152.07
7	153.56
8	154.17
9	195.60
10	153.87
11	202.31
12	147.14
13	157.63
14	154.78
15	153.84
